# Diverse Shape Design and Physical Property Evaluation of In-Body Tissue Architecture-Induced Tissues

**DOI:** 10.3390/bioengineering11060598

**Published:** 2024-06-12

**Authors:** Tsutomu Tajikawa, Yota Sekido, Kazuki Mori, Takayuki Kawashima, Yumiko Nakashima, Shinji Miyamoto, Yasuhide Nakayama

**Affiliations:** 1 Department of Mechanical Engineering, Faculty of Engineering Science, Kansai University, Osaka 564-8680, Japan; 2 Graduate School of Science and Engineering, Kansai University, Osaka 564-8680, Japan; k743522@kansai-u.ac.jp; 3 Department of Cardiovascular Surgery, Oita University Hospital, Oita 879-5593, Japan; kazumori@oita-u.ac.jp (K.M.); t-kawashima@oita-u.ac.jp (T.K.); nakashima-y@oita-u.ac.jp (Y.N.); smiyamot@oita-u.ac.jp (S.M.); 4 Osaka Laboratory, Biotube Co., Ltd., Osaka 565-0842, Japan; y.nakayama@biotube.co.jp

**Keywords:** in-body tissue architecture, tissue engineering, biofabrication, implantable tissue, aortic graft with branching, long cord-shaped tissue, mechanical strength

## Abstract

Autologous-engineered artificial tissues constitute an ideal alternative for radical surgery in terms of natural anticoagulation, self-repair, tissue regeneration, and the possibility of growth. Previously, we focused on the development and practical application of artificial tissues using “in-body tissue architecture (iBTA)”, a technique that uses living bodies as bioreactors. This study aimed to further develop iBTA by fabricating tissues with diverse shapes and evaluating their physical properties. Although the breaking strength increased with tissue thickness, the nominal breaking stress increased with thinner tissues. By carving narrow grooves on the outer periphery of an inner core with narrow grooves, we fabricated approximately 2.2 m long cord-shaped tissues and net-shaped tissues with various designs. By assembling the two inner cores inside the branched stainless-steel pipes, a large graft with branching was successfully fabricated, and its aortic arch replacement was conducted in a donor goat without causing damage. In conclusion, by applying iBTA technology, we have made it possible, for the first time, to create tissues of various shapes and designs that are difficult using existing tissue-engineering techniques. Thicker iBTA-induced tissues exhibited higher rupture strength; however, rupture stress was inversely proportional to thickness. These findings broaden the range of iBTA-induced tissue applications.

## 1. Introduction

Autologous tissue-engineered artificial tissues for blood vessels or heart valves are considered to be the ideal alternative tissues for radical surgery in terms of natural anticoagulation, self-repair, tissue regeneration, and adaptability to growth [1,2]. To realise these ideal artificial tissues, in vivo tissue-engineering technologies that use living bodies as incubators are attracting attention from the viewpoints of simplicity and safety.

Our research group has focused on the development and practical application of artificial tissues through “in-body tissue architecture (iBTA)”, which is an in vivo tissue-engineering approach [3,4]. If foreign substances with low inflammatory potential are present subcutaneously, fibroblasts accumulate around the foreign substance to form collagen. This results in the formation of a capsule comprising a collagen-rich extracellular matrix that encapsulates the foreign substance [5,6]. This encapsulation phenomenon is well known, and iBTA is a practical concept and method for in vivo tissue engineering that exploits this phenomenon to create a biochemically or morphologically designed environment and scaffold for histogenesis for stable cell derivation and engraftment [3]. We have previously applied iBTA to fabricate 2D and 3D structures such as tubular or membranous tissues and their combination, not only autologously but also allogeneically or xenogeneically without donor sacrifice. These tissues were subjected to the following applications: vascular grafts (Biotubes) for the carotid or aortic artery with a diameter of 0.6–5.0 mm [7,8,9,10,11,12,13,14] or shunt grafts for dialysis [15], aortic stent grafts [16,17], and aortic, pulmonary, or mitral heart valves (Biovalves) [18,19,20,21,22,23,24,25,26,27,28,29]. Patches for the peritoneum [30], dura mater [31], trachea [32,33], and skin grafts [31] have also been developed. The mechanical properties of these tissues have been investigated using a tensile tester [34], and the haemodynamic performance of artificial blood vessels and heart valves has been evaluated using in vitro experiments [35] and computer simulations [36], such as computer fluid mechanics and finite element structural analysis. We also conducted experiments and evaluated the effectiveness of these engineered tissues by transplanting them into experimental animals.

The aim of this study was to apply iBTA to fabricate tissues of various shapes and sizes that had previously been impossible to create artificially. We aimed to fabricate ring-shaped tissues of various thicknesses, cord-shaped tissues, and net-shaped or large-branched tissues by adjusting the mould design to meet individual requirements. The mechanical properties of the obtained tissues were investigated.

## 2. Diverse Shaped iBTA-Induced Tissues

### 2.1. Ethical Approval

All animals were maintained under the “Guide for the Care and Use of Laboratory Animals” published by the US National Institutes of Health (NIH Publication No. 85-23, revised 1996). The research protocol was approved by the Oita University Animal Ethics Committee (Protocol no. 1822001) and NAS Laboratory Co., Ltd. (Protocol no. 22 L-G061, 22 L-G076, and 23-G022).

### 2.2. Fundamental of Moulds for Preparation of iBTA-Induced Tissues

**Figure 1:**
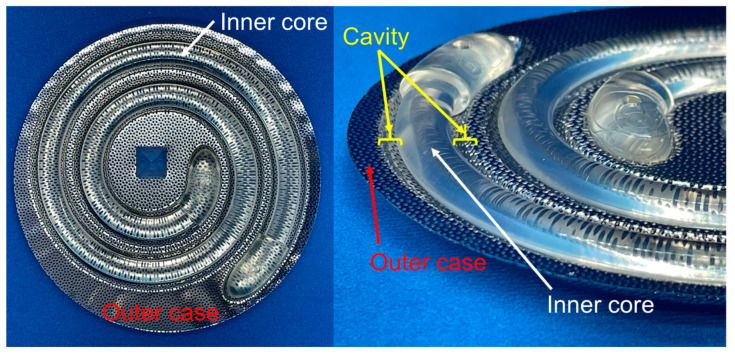
An example of a mould for in-body tissue architecture (iBTA)-induced tissue fabrication (Biotube maker). The mould is a combination of a stainless-steel outer case with a resin inner core. The iBTA-induced tissue is generated in the cavities located between the outer case and the inner core.

The basic structure of the moulds for fabricating iBTA-induced tissues is shown below. The moulds primarily consisted of outer cases with micropores and inner cores of several geometries [8,9,13,30,34]. Figure 1 shows a typical example of a mould (Biotube maker). The outer case has numerous pores that allow cells to be introduced into the cavity between the outer case and the inner core, which are then filled with a collagen-rich extracellular matrix.

### 2.3. Fabrication Method for Ring-, Rod-, or Net-Shaped iBTA Tissues

**Figure 2:**
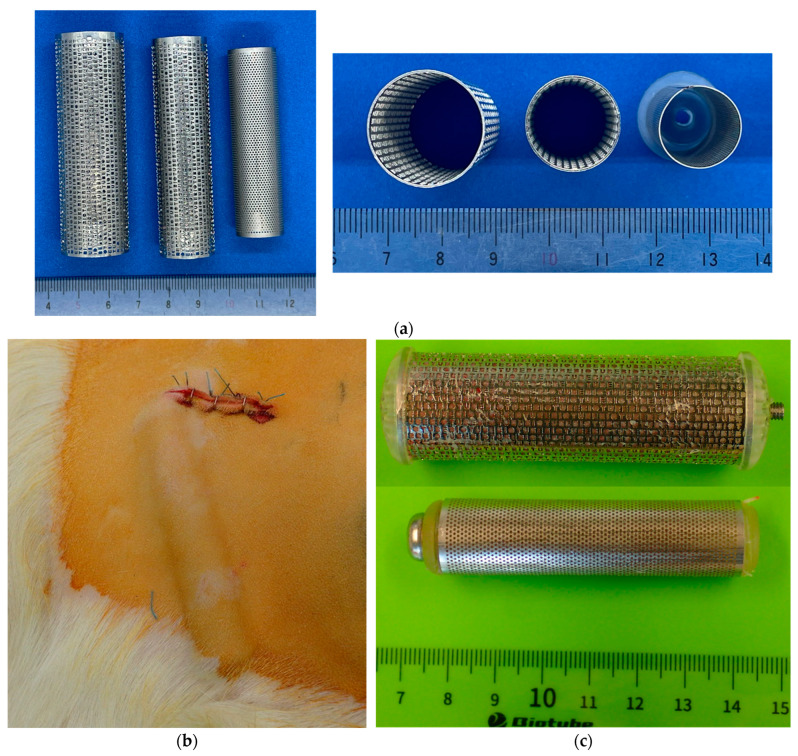
Images of in-body tissue architecture-induced tissue moulds used in this study. (**a**) Stainless steel circular conduits for the outer case. (**b**) Mould subcutaneously embedded in a goat. (**c**) Typical examples of harvested moulds.

To fabricate ring- or rod-shaped iBTA tissues, we designed a cylindrical mould shown in Figure 2a. The outer cases were electrolytically polished stainless-steel pipes with circular or ellipsoidal cross-sectional shapes with a diameter of 10–25 mm, length of 60–150 mm, and wall thickness of 0.3–0.5 mm. Pores were introduced by chemical etching or laser machining, with a diameter of 0.1–1.0 mm, to enable cellular communication between the cavities and the outside of the moulds. The pore shape, size, pore layout, and total pore number were not the same as their outer moulds, and the aperture ratio of the surface of the porous mould was designed to be approximately 30–40%.

All pre-assembled moulds were autoclaved and embedded subcutaneously in the abdomens of 10 Saanen goats (age = ≥12 months, weight = 39–70 kg, 4–8 moulds per animal), as shown in Figure 2b. For anaesthesia, induction was conducted using 2 mg/kg ketamine and maintenance with 2–3% sevoflurane. After 8 weeks of embedding, the moulds were harvested under general anaesthesia, and the tissues extracted from the moulds were stored temporarily in a 70 wt% ethanol solution for 30 min and then preserved in a 10% ethanol solution. During the preparation of the animal implantation procedure, the fabricated tissues, such as the Biotubes, were rinsed with a saline solution for 10 min. As the measurements of the mechanical properties of the tissues were performed on another day, the tissues were preserved in a saline solution containing 10 wt% alcohol.

Figure 2c shows typical examples of moulds soon after harvesting from the subcutaneous tissue of goats. According to the externality estimation, no damage was observed during subcutaneous embedding at the abdomen. In addition, no close contact was observed between the other moulds and subcutaneous tissue without liquid retention around the moulds while embedding. None of the goats showed mould inflammation, infection, exposure, or any signs of distress after the predetermined embedding procedure. As there were very few inflammatory cells in the histological images of the tissue produced using iBTA in previous studies [8,37], the primary reaction during the tissue production was not inflammation but the encapsulation of foreign material. All moulds were firmly attached to the subcutaneous tissue, with subcutaneous tissue infiltration into the cavities of the moulds occurring through the micropores. The details of the inner core and harvested tissue from each mould are shown in the subsequent sections. The composition of our fabricated tissues is currently being investigated on a sufficient number of samples, along with the relationship with mechanical properties. Therefore, these results will be reported in a separate study in the future. However, few histological images showed that the harvested connective tissues mainly comprised collagen produced by fibroblasts [3,4,7,9] and that the compact layers of collagen in the tissue were likely located in the vicinity of the surface [8]. These were not different from those of previous studies.

### 2.4. Ring-Shaped iBTA Tissues and Their Mechanical Properties

**Figure 3:**
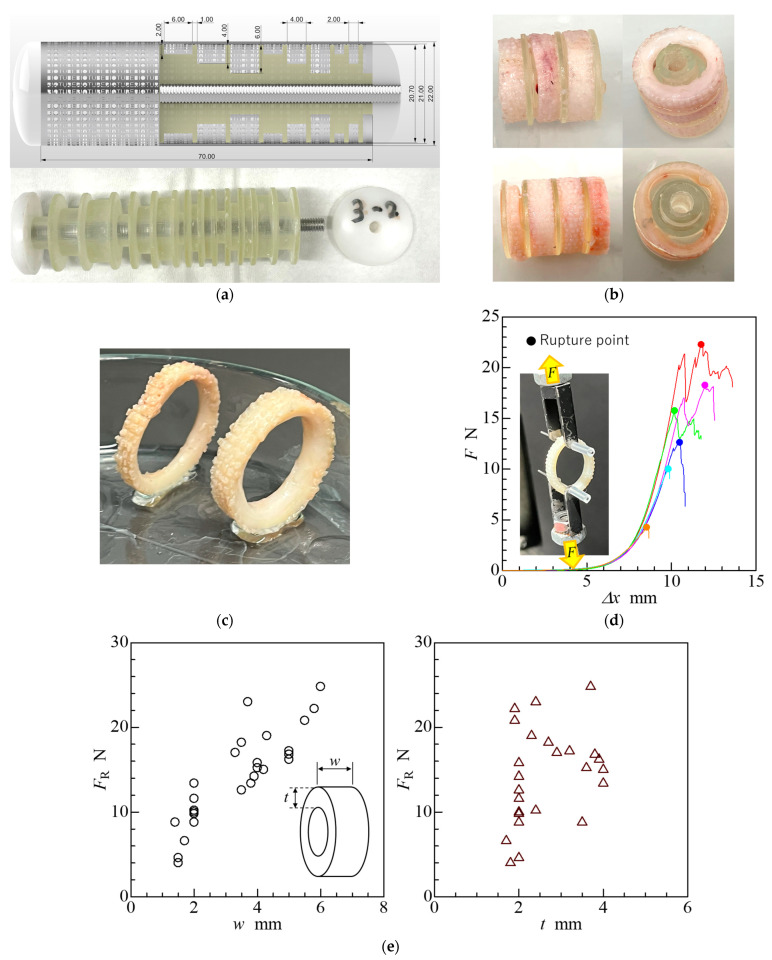

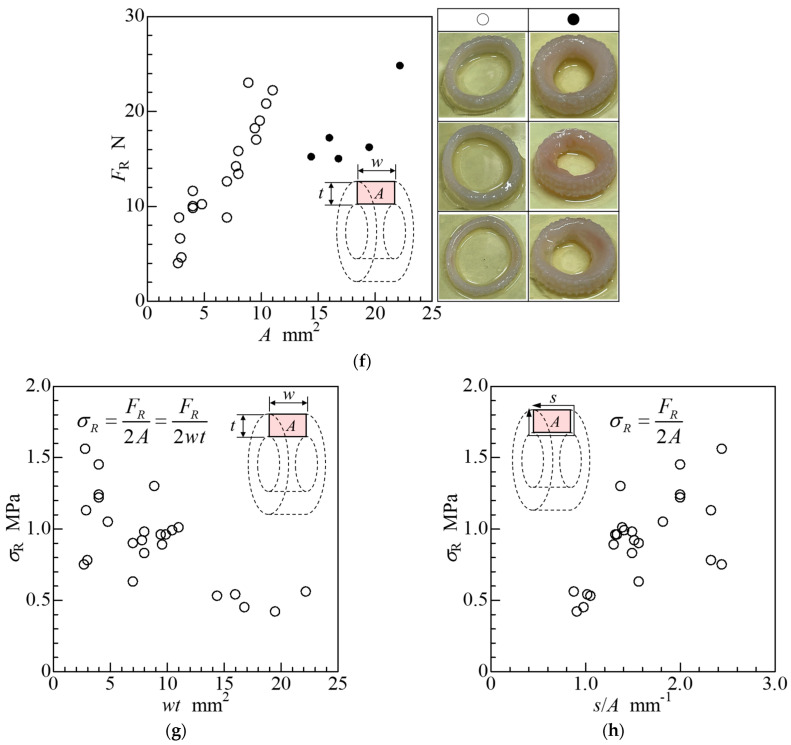
Thicker ring-shaped tissue. (**a**) Image of the inner core and computer graphic of the cross-sectional view of the mould. (**b**) Harvested ring-shaped tissues with inner cores. (**c**) An example of 4 mm thick tissue that did not collapse under its own weight. (**d**) Six examples of displacement (*Δx*)–force (*F*) curves. (**e**) Influence of tissue width (*w*) and thickness (*t*) on the tensile rupture strength (*F*_R_) of ring-shaped tissue. (**f**) Relationship between the rupture strength and cross-sectional area (*A*) of harvested tissue. (**g**) Relationship between nominal tensile stress (*σ*_R_) at each rupture point and cross-sectional area of the tissue. (**h**) Relationship between rupture stress and the ratio of perimeter(*s*) and cross-sectional area of the tissue.

To investigate the feasibility of fabricating thicker structures with higher rupture strengths, we designed and fabricated moulds to produce ring-shaped tissues with thicknesses of 2, 4, and 6 mm, using a cylindrical outer case with an outer diameter of 22 mm, inner diameter of 21 mm, and length of 70 mm, as shown in Figure 3a. The outer stainless-steel cases were porous with an equivalent pore diameter of approximately 0.75 mm; the inner cores were inserted into the outer cases. The inner cores were composed of stacked cylinders fabricated using a 3D printer (Form3B+; Formlabs, Somerville, MA, USA) and were made from photocrosslinkable resin (Clear Regin; Formlabs). Each cylindrical inner core had different outer diameters and heights and a dividing plate, with an outer diameter of 20.7 mm and a height of 1 mm, at its end to separate the neighbouring connective tissues. The stacked cylindrical cores were fixed in the outer cases by inserting a stainless-steel-threaded rod into a hole in the centre of the cylindrical inner cores and screwing them using machined polyacetal (POM) caps at both ends of the outer cases.

Figure 3b shows examples of the extracted ring-shaped tissues. Almost all cavities in the moulds were filled with connective tissue, but the tissue did not sufficiently infiltrate some cavities with a depth of 6 mm. If there is an upper limit to the total amount of collagen produced by a fibroblast cell, deep mould cavities would be difficult to fill with connective tissue under conditions of the same porous aperture area. The size and quantity of micropores in the outer case were considered to impact cell conductance through the micropores. We employed porous outer cases with sufficiently large aperture areas. However, if the micropores are occluded by the collagen produced before a sufficient amount of subcutaneous tissue migrates through the micropores into the cavities, the cavities would not be filled with connective tissue [9]. Therefore, the maximum thickness of the tissue that could be fabricated by iBTA was approximately 5–6 mm, depending on the inner core material. The ratio of the total pore opening area in the outer case to the volume of the cavities was considered an important factor for ensuring connective tissue fabrication.

As shown in Figure 3c, tissues with a thickness over 2 mm were not deformed by their weight. Therefore, thicker tissues might be sufficiently stiffer to maintain their shape. Ring-shaped tissues were subjected to uniaxial tensile tests to evaluate their mechanical properties using a uniaxial tensile tester (Autograph; Shimadzu, Kyoto, Japan). The tensile force at which the samples were pulled and broke was recorded as the breaking strength. Additionally, the tensile force was defined as the load applied to a ring-shaped tissue [38]; it can be measured directly using the tensile tester. The method for conducting the strength test was according to a previous study [8]. All tensile tests were performed on tissues preserved in an alcohol–saline solution and not on fresh tissue. Therefore, before the tensile testing, the preserved tissues were rinsed with saline solution for 10 min.

Examples of the displacement (Δ*x*)–force (*F*) curves obtained during tensile testing are shown in Figure 3d, which shows that the mechanical properties of ring-shaped tissue exhibit a load-displacement relationship with a J-shaped curve [39], similar to that of typical biological tissues. The tensile force at the rupture point was determined as the rupture strength (*F*_R_) from these graphs, and the relationship between the rupture strength and tissue size (width: *w*, thickness: *t*) is shown in Figure 3e. These results showed that the rupture strength tended to be proportional to the tissue width, but the tissue thickness remained unaffected. The relationship between the rupture strength and cross-sectional area (*A*) of the tissue before tensile testing is shown in Figure 3f.

The results showed that the increase in rupture strength is directly proportional to the cross-sectional area of the tissue. However, when the cross-sectional area of the tissue exceeded approximately 12 mm^2^, the rupture strength abruptly decreased to approximately 16 N. Tissues with a cross-sectional area of more than 12 mm^2^ were harvested from inner cores with a cavity depth of 6 mm. The outer shape of the ring-shaped tissue was entirely transcribed by the lumen of the outer case; however, the luminal surface of the ring-shaped tissue was not the same as that of the inner core. Therefore, the surface area of the tissue in contact with the inner core may affect rupture strength.

When connective tissue forms and grows in the moulds, a foreign-body reaction is presumed to occur strongly near the surface of the synthetic resin, and compact layers of collagen in the tissue are likely located in the vicinity of the surface [8]. As the surface area per unit volume of each tissue is smaller in large tissues than in small tissues, the amount of the compact collagen layer in the tissue is insufficient to impart strength. The relationship between the nominal tensile stress at the rupture point (*σ*_R_), which is defined as the ratio of the rupture strength (*F*_R_) to twice the cross-sectional area (2*A*) of the tissue before tensile testing, is shown in Figure 3g. The relationship between nominal stress and average tissue thickness (*A*/*s*), which is obtained by dividing the tissue cross-sectional area by its cross-sectional perimeter (*s*), is shown in Figure 3h. These results indicate that the nominal rupture stress of the tissue tends to be inversely proportional to the cross-sectional area and proportional to the reciprocal of the average tissue thickness (*s*/*A*). These results indicate that an inner core with a large surface area that allows for a large number of compact collagen layers is necessary to produce a tissue with a high rupture strength.

### 2.5. Cord-Shaped iBTA Tissues and Their Mechanical Properties

We fabricated various 3D connective tissues using iBTA [31]. However, the mechanical properties of the tissues have not yet been sufficiently controlled in any way other than by changing their thickness. Therefore, investigating other tissue fabrication methods from the above-mentioned conventional methods to control the mechanical properties of the regenerated tissue produced is necessary.

As mentioned in Section 2.3, thinner ring-shaped tissues are susceptible to higher rupture stress. To produce large tissues with high rupture strength, we considered fabricating large tissues assembled from numerous bundled thinner tissues rather than direct large-tissue fabrication from a mould to be more effective. Therefore, we propose that one solution is to combine cord-shaped tissues, which can easily be produced in large quantities under the same conditions. As the first step in this novel fabrication method, we designed moulds that could produce thin and fine cord-shaped tissues.

**Figure 4:**
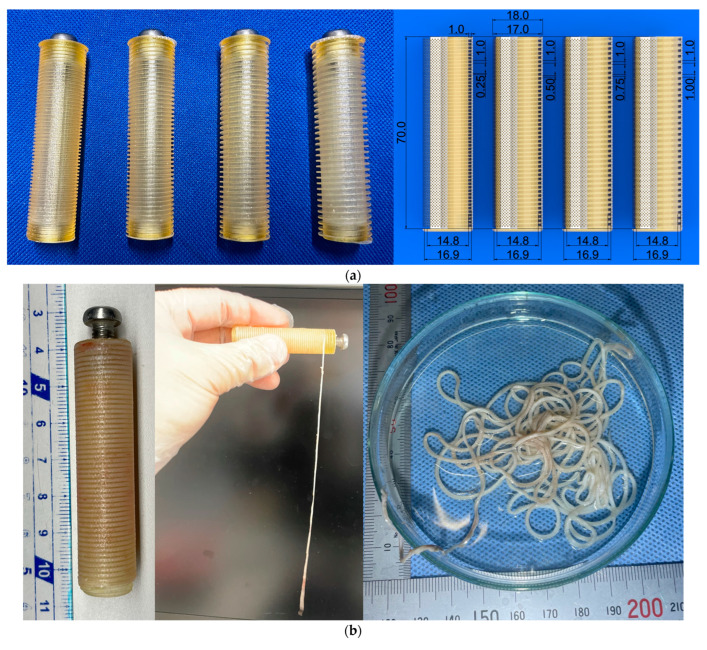

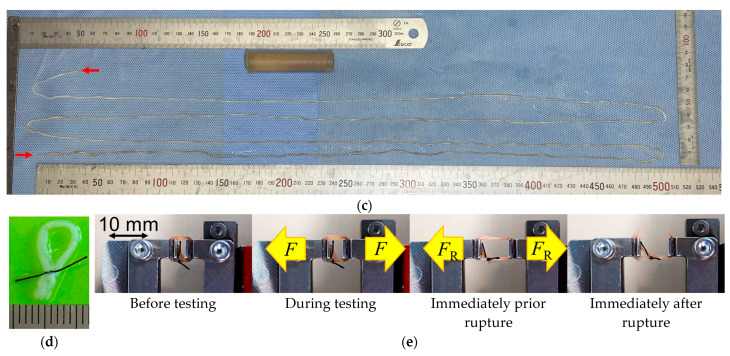
Cord-shaped tissue. (**a**) Image of the inner core and its dimensions. (**b**) Image of a harvested inner core with the generated tissue (**left**), cord-shaped tissue extraction from the mould (**middle**), and extracted cord-shaped tissue (**right**). (**c**) Over 2 m of autologous tissue-engineered artificial tissue was obtained. The red arrows show both ends of the cord-shaped tissue. (**d**) An example of the tensile test sample for cord-shaped tissue. (**e**) Images at pre-loading, during loading, immediately prior to rupture, and immediately after rupture.

Examples of fine cord-shaped tissue moulds are shown in Figure 4a. The inner core of the cord-shaped tissue is designed as a groove with a cross-sectional area of 1 mm. The groove is located helically at 0.25–1.0 mm intervals, similar to square threads, and the total length of the groove in the inner core, of which the axial length and outer diameter of the outer case were 70 and 17 mm, respectively, was approximately 1.7–2.7 m. If collagen were filled not only in the groove on the inner core but also in the gaps between the core and outer case, it would become difficult to remove the tissue from the mould because the cord-shaped tissues would be connected by a thinner collagen film generated by fibroblasts. Therefore, the gap between the outer lumen and the outer surface of the inner core was designed to be less than 0.05 mm.

The outer cases were made from seamless stainless-steel tubing, which was thin-walled by lathing and made porous by laser drilling. The inner cores were fabricated using light-curing resin (AR-M2, Transparent resin; Keyence, Osaka, Japan) in a 3D printer (Agilista-3200, Keyence, Osaka, Japan). However, unlike the inner core of the aortic arch graft, the grooves where the tissue formed were narrow; therefore, the surfaces of the grooves were not polished.

As shown in Figure 4b,c, we were able to fabricate cord-shaped tissue with a square cross-section of 1.0 mm^2^ and a longitudinal length of over 2 m. To the best of our knowledge, this piece of cord-shaped tissue is currently the world’s longest autologous tissue-engineered artificial tissue.

In the cylindrical outer case used in the current study, the length of the harvested tissue increased proportionately with the outer diameter and length of the mould. In our previous experiments, we embedded cylindrical moulds with an inner diameter of 21 mm and a length of 100 mm, which would theoretically yield cordage of approximately 4 m. If numerous long cord-shaped tissues can be obtained, producing cloth would be possible. Furthermore, this method is expected to produce cloth made from collagen-based cord-shaped self-organised tissue. The rupture strength is also expected to improve by bundling several strings together. As the mechanical strength of the bundled cord-shaped tissues could be stiffer, this cord-shaped tissue could be applied to tendon/ligament grafts.

A mechanical strength test was conducted on the cord-shaped tissues using a uniaxial tensile tester (Stency; AcroEdge, Osaka, Japan). The method employed in the strength test was similar to that described in Section 2.2. The cord-shaped tissues with 1 mm square cross-sectional shapes were cut into 20–30 mm in length. Every sample for the tensile test was made into a loop, and a ligature was applied using a silk surgical suture thread (size: 4/0) at approximately 5 mm away from both ends of a cord, as shown in Figure 4d. Figure 4e shows an example of the sample’s behaviour under a tensile test. The strength test revealed that the rupture strength (*F*_R_) and stress (*σ*_R_) of the cord-shaped tissue were approximately 4.16 ± 0.47 N and 2.08 ± 0.23 MPa (*n* = 10), respectively. According to a previous study [40], the rupture stress of tendon-like tissue generated from human induced pluripotent stem cells was approximately 0.5 MPa; therefore, the rupture stress of the fabricated cord-shaped tissue was approximately 4-fold higher. These cord-shaped tissues have the potential for use as artificial tendons. However, human medial collateral ligaments have a rupture stress of approximately 50 MPa [41], which is 25-fold higher than that of iBTA-induced cord-shaped tissue. Therefore, harvesting thinner cord-shaped tissue and denser compact layers of collagen is necessary to further improve mechanical strength.

### 2.6. Net-Shaped iBTA Tissues

**Figure 5:**
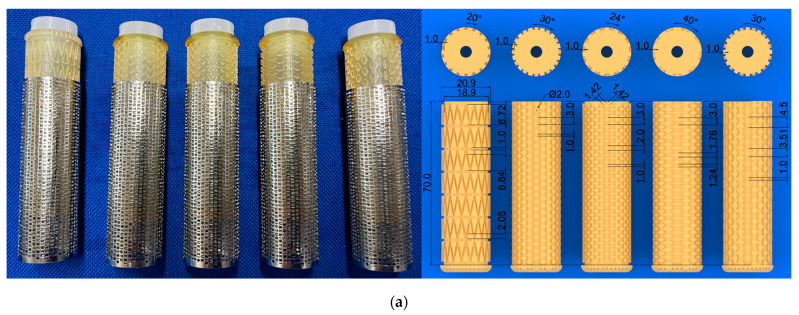

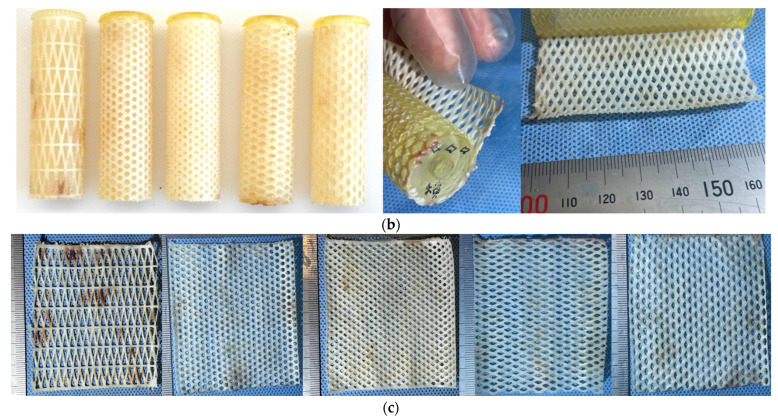
Meshed tissue. (**a**) Image of inner cores assembled into the outer cases (**left**) and their dimensions (**right**). The images and illustrations are shown in the order starting from left: truss mesh, circular pore, square pore, circumferential long rhombic, and axially long rhombic. (**b**) Image of harvested inner cores with generated tissues (**left**) and meshed tissue extracted from the inner core (**middle** and **right**). (**c**) Images of the fabricated tissues. The order of the images is the same as that in (**a**).

Thinner cord-shaped tissue will be made into cloth by weaving or thicker by bundling; however, this is not efficient in terms of fabricating 3D tissue. Therefore, we considered obtaining thin sheet-like tissues with various patterns that could be constructed by combining several cord-shaped tissues directly from a mould. Therefore, we propose that one solution is to combine net-shaped tissues. An example is shown in Figure 5a. The outer cases were the same as those of the ring-shaped tissue. To obtain net-shaped tissue, numerous pillars with a height of 1 mm were placed on the surface of the inner core. The cross-sectional shapes of the pillars were triangular, circular, square, or rhombic. The gap between the pillars was approximately 1.0–1.5 mm. Similar to the inner core for cord-shaped tissues, the clearance between the inner core and lumen of the outer case was designed to be 0.05 mm. In addition, the inner core was designed such that the wire width of the mesh was 1 mm, and the opening area of the mesh was approximately 10 mm^2^ with a triangular opening, as shown in Figure 5a (far left). Four additional inner cores were designed such that the opening area of the mesh was approximately 2 mm^2^, and the opening shapes were circular, square, and rhombus. The inner cores were fabricated using light-curing resin (AR-M2, Transparent resin; Keyence, Osaka, Japan) in a 3D printer (Agilista-3200; Keyence, Osaka, Japan) and used without polishing.

Figure 5b,c show the harvested inner core and tissues extracted from the mould. In this experiment, a mesh with a minimum cord diameter of 1 mm and a minimum mesh opening area of 2 mm^2^ was fabricated. By combining cords and net-shaped tissues, tissues with various shapes and high strengths are expected to be produced. Although a uniaxial tensile test was not conducted for net-shaped tissue, it was palpably stiffer than cord-shaped tissues and sufficiently flexible. This suggests that net-shaped tissue can be used as a reinforcement material for artificial blood vessels and patches.

### 2.7. Branch-Shaped iBTA Tissues

**Figure 6:**
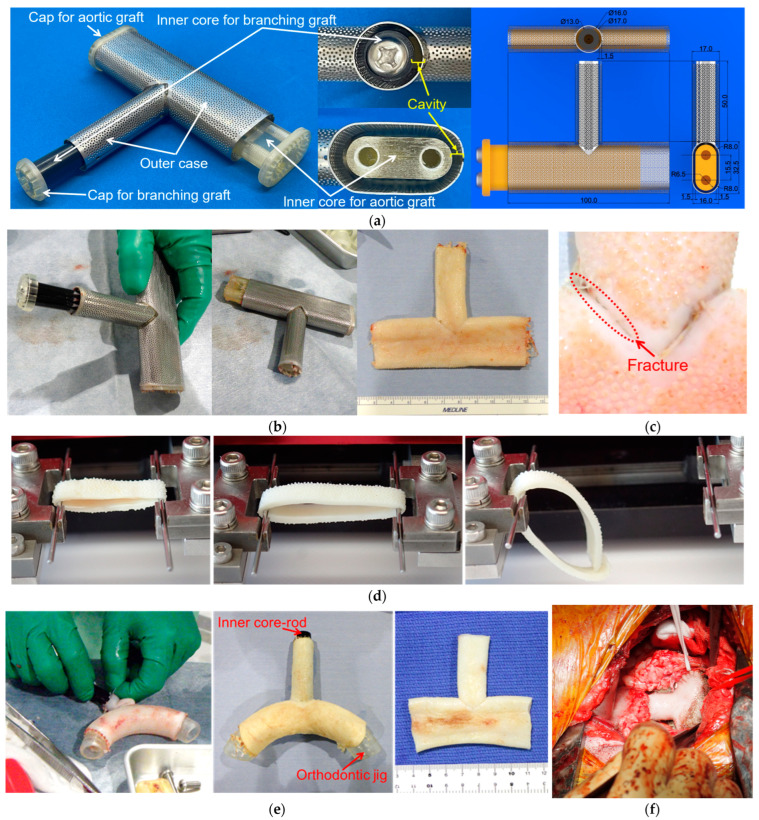
T-shaped Biotube. (**a**) Image of the T-shaped Biotube mould and its dimensions. (**b**) Extracted T-shaped Biotube from the mould. (**c**) An example of an initially fractured T-shaped Biotube. (**d**) Branching graft section (**left**) and parent vessel section (**middle**) during uniaxial tensile test and immediately after rupture of the parent vessel section (**right**). (**e**) Using a 3D-printed orthodontic jig, a straight aortic graft imparted a banana-like curvature. (**f**) Image of aortic arch replacement in a donor goat.

A mould for a one-piece graft with both the aortic arch and its branching artery (hereafter referred to as a T-shaped Biotube) was designed and prepared, as shown in Figure 6a. This T-shaped Biotube was designed by mimicking the vascular morphology of goats to evaluate their utility in goats, which are almost the same size as adult humans. As the native aortic arch is curved, fabricating curved outer cases and cylindrical inner cores with high precision is challenging. Therefore, in the present study, we designed the aortic arch graft as a straight tube. The morphology of our fabricated T-shaped Biotube was, therefore, different from human aortic arch grafts, which are already in clinical use. The size of the T-shaped Biotubes was determined based on thoracic computed tomography data from several goats. The parent vessel had an inner diameter of 23 mm, a wall thickness of 1.5 mm, and a length of 100 mm. A branching graft (13 mm inner diameter, 1.5 mm wall thickness, and 50 mm axial length) was placed in the middle of the aortic arch graft perpendicular to the axis of the aortic arch.

There were concerns that the combination of outer cases of different diameters would create gaps between the outer case surface and the subcutaneous tissue under the skin, which would slow tissue generation by reducing the conductivity of cell migration from the subcutaneous tissue to the outer case surface. Further concerns included fears that the gap would induce inflammation that would erode the epithelial tissue at the edges of the gap. To prevent subcutaneous gap formation after mould embedding, we designed the height of the outer case of the aortic arch graft to be the same as the outer diameter of the outer case of the branching graft. Therefore, the cross-sectional shape of the outer case of the aortic arch was not circular but oval, with the same perimeter as that of a circular tube with an inner diameter of 23 mm and a thickness of 1.5 mm.

The outer mould for the aortic arch graft was fabricated from a chemically etched porous stainless-steel sheet with a 0.5 mm thickness, followed by tungsten inert gas (TIG) welding to produce a circular sealed pipe. The external mould used for the aortic arch graft was obtained by deformation from a circular to an oval shape using pressure. The outer cases of the aortic arch and branching grafts were bonded by TIG welding, and their inner surfaces were thoroughly polished before use.

After mould harvesting, a one-piece aortic arch graft with bifurcation was extracted from the mould without damaging it. The inner core of the aortic arch graft and the inner core of the branching graft were fabricated separately, and the cores were assembled and fixed to the outer case using screws and caps to form gaps, with uniform clearance between the cores and the outer case.

Owing to the ease of prototyping, the inner-core rod of the aortic arch graft was fabricated using light-curing resin (AR-M2, Transparent resin; Keyence, Osaka, Japan) in a 3D printer (Agilista-3200; Keyence, Osaka, Japan). The surface of the printed inner-core rod was thoroughly polished before use. The inner-core rod of the branching graft comprised a circular tube made from POM, owing to its smoother surface without extra polishing when compared with that of 3D-printer products. Using the assembled inner-core rods, a one-piece graft was extracted without damaging the moulds.

As shown in Figure 6b, by disassembling the inner cores, a T-shaped Biotube was successfully fabricated and extracted from the mould without damage. However, as shown in Figure 6c, some of the harvested Biotubes initially had eccentric tubes and fractures. These problems were primarily due to the insufficient accuracy of the oval cross-sectionally shaped outer case. Therefore, for reliable coaxial centring between the inner core and conduit-shaped outer case, it is necessary to assume that both the inner core and the outer case have circular cross-sections and are arranged in concentric circles. T-shaped Biotubes were subjected to uniaxial tensile tests, as shown in Figure 6d. The method and a tensile tester employed in the strength test were similar to that described in Section 2.5. The tensile test samples were obtained from the parent vessel grafts and the branching grafts, and each sample was cut with a 5 mm width. The strength test revealed that the rupture strength of the tubular tissue was approximately 26.2 ± 8.2 N at a parent vessel graft (*n* = 15) and 33.3 ± 3.4 N at a branching graft (*n* = 6). The mean rupture strength of the Biotube for ascending aorta replacement was approximately 22 N, and no Biotube fracture was observed during the 6-month observation period after replacement [42]. This observation suggests that T-shaped Biotubes can be used for aortic arch replacement.

To demonstrate feasibility, aortic arch replacement in a donor goat was conducted using a T-shaped Biotube immediately after harvesting. To alter the Biotube into a banana-like curve through slight dehydration and shrinking, the harvested Biotube was temporarily stored in 70% ethanol solution for 30 min after inserting a 3D-printed orthodontic jig, as shown in Figure 6e. The Biotube was then rinsed with a saline solution for 10 min. Figure 6f shows an image of the replaced aortic arch immediately after the heart–lung machine was removed from the goat. The Biotube demonstrated potential in the aorta, which is a tough environment subjected to very high stress, owing to the large vessel diameter and high blood pressure. To evaluate the performance and effectiveness of the T-shaped Biotube, quantitative evaluation through implantation experiments and long-term follow-up is required. Therefore, we are continuing long-term follow-up after implantation and increasing the number of replacement cases using the T-shaped Biotube and will report the results in a future study.

Since the influence of differences in the materials used in iBTA-induced tissue moulds on the mechanical properties of the harvested tissue is unclear, we have planned to fabricate tissues using various resins and evaluate their mechanical properties. In addition, we will attempt to produce smaller Biotubes with bifurcations. Furthermore, to generate higher-strength tissues, we intend to design and fabricate moulds with larger surface areas and shallower cavities for tendon grafting.

## 3. Conclusions

This study describes iBTA-induced tissues, including thicker tissue, grafts for large, branched vessels, the world’s longest tissue produced in vivo, and net-shaped tissues. Thicker iBTA-induced tissues had a higher rupture strength, but the rupture stress was inversely proportional to the thickness. These results are expected to expand the range of applications of iBTA-induced tissues.

## Data Availability

The data are not publicly available because they contain information that can compromise the privacy of the research participants.
